# Microfluidic-based high-throughput isolation enhances the recovery of novel strains and diversity from Arctic soil microbiome

**DOI:** 10.1128/aem.01124-26

**Published:** 2026-09-29

**Authors:** Li Liao, Tingyi Lai, Wanning Jiang, Zedong Duan, Peiyuan Sun, Yunpeng Zhao, Fang Peng, Qilong Qin, Mukan Ji, Siqi Zhang

**Affiliations:** 1 National Arctic and Antarctic Data Center, Ministry of Natural Resources, Polar Research Institute of China 127281, Shanghai, China; 2 Key Laboratory for Polar Science, Ministry of Natural Resources, Polar Research Institute of China, Shanghai, China; 3 School of Oceanography, Shanghai Jiao Tong University 722366, Shanghai, China; 4 TMAXTREE Biotechnology Co., Ltd. 267439 , Wuxin, China; 5 China Center for Type Culture Collection (CCTCC), College of Life Sciences, Wuhan University 98436, Wuhan, China; 6 State Key Laboratory of Microbial Technology, Shandong University 214177, Qingdao, China; 7 Center for Pan-third Pole Environment, Lanzhou University 12426, Lanzhou, China; 8 School of Medical Instrument and Food Engineering, University of Shanghai for Science and Technology 47863, Shanghai, China; University of Delaware, Lewes, Delaware, USA

**Keywords:** Arctic soil, microfluidic droplet technology, microbial isolation, PacBio sequencing

## Abstract

Microbial cultivation remains essential for understanding the physiology, ecology, and biotechnological potential of environmental microbes, yet conventional plate-based methods (CPM) recover only a minute fraction of the environmental microbiome. Polar regions, particularly Arctic soils, represent unique reservoirs of “microbial dark matter” that remain challenging to cultivate, owing to oligotrophic conditions, low temperatures, and freeze-thaw cycles that impose severe physiological constraints on microbial growth. Here, we report the first systematic application of microfluidic droplet technology (MDT) to Arctic active-layer soil microbiota and benchmark its performance against CPM using identical starting cell numbers, R2A medium, and incubation at 15°C. MDT achieved 6.5- to 8.1-fold higher recovery rates than CPM and improved isolation throughput by >180-fold. Near-full-length 16S rRNA gene sequencing (PacBio) revealed that MDT recovered significantly higher taxonomic richness across all taxonomic levels, with 256 genera detected in the high-cell-input group (DropAS_H) versus 211 in the corresponding plate group (PlateAS_H). Notably, MDT yielded a more even community distribution, significantly reducing the dominance of fast-growing copiotrophs, such as *Pseudomonas* and *Flavobacterium*. Moreover, approximately 50% of the sequences from MDT were affiliated with potential novel species (<98.65% identity to type strains), and 27% with potential novel genera (<95% identity). Strain verification by Sanger sequencing confirmed 12 of 17 isolates as candidate novel species, among which one strain represented a potential novel genus within *Devosiaceae*. This study demonstrates that MDT is a powerful platform for accessing the uncultured majority of polar soil microbiota and establishes a pipeline for high-throughput isolation of novel cold-adapted bacteria.

**IMPORTANCE:**

Arctic soils harbor a vast reservoir of microbial diversity that remains largely inaccessible due to the extreme oligotrophic conditions and low temperatures characteristic of polar environments, leading to slow growth and extended lag phases in most microbes. Conventional plate-based methods (CPM) inherently favor fast-growing copiotrophs while suppressing rare or slow-growing lineages. Here, we demonstrate that microfluidic droplet technology (MDT) overcomes these fundamental constraints, representing its first systematic application to polar microbiology. By physically isolating individual cells into microliter-scale bioreactors, MDT provides independent microenvironments that allow slow-growing and oligotrophic taxa to proliferate without being outcompeted by fast-growing copiotrophs in bulk cultures. The water-in-oil emulsion format further enables extended low-temperature incubation without evaporative loss or airborne fungal contamination, issues that frequently compromise long-term plate-based cultivation of Arctic samples. Relative to CPM, MDT increased recovery rates by over 6-fold and isolation throughput by 180-fold, while markedly enhancing both taxonomic richness and evenness. Exclusively recovered by MDT, the oligotrophic genus *Caulobacter* and numerous cold-adapted genera underscore that MDT accesses physiologically distinct fractions of the cryospheric microbiome. Furthermore, the integration of near-full-length 16S rRNA gene sequencing with MDT cultivation assessment provided substantially improved phylogenetic resolution for species-level identification and novel taxon delineation. Collectively, these findings establish MDT as a transformative platform for cryospheric culturomics, accelerating the construction of comprehensive polar strain collections essential for understanding cold-adaptation mechanisms and exploiting the biotechnological potential of Earth’s frozen microbiomes.

## INTRODUCTION

It is widely recognized that less than 1% of environmental microbes are readily culturable using conventional plate-based methods (CPM), a phenomenon termed the “Great Plate Count Anomaly” (1). The remaining uncultured species constitute “microbial dark matter” that awaits discovery (2, 3). Although culturability can exceed 1% under certain circumstances using improved methods (4, 5), the majority of species remain uncultivated (6), especially given the continuously expanding diversity revealed by high-throughput deep sequencing (7). Based on 16S rRNA gene sequencing, approximately 400,000 prokaryotic species comprising about 60,000 genera have been detected (6, 8). Despite this immense diversity, most cultivated bacterial species belong to only four bacterial phyla: *Bacteroidota*, *Pseudomonadota*, *Bacillota*, and *Actinomycetota* (6), highlighting a profound cultivation bias. While advanced single-cell sequencing and multi-omics have expanded our understanding of uncultured microorganisms (9), the limited success of cultivation greatly hinders in-depth investigation of physiology, phenotypes, real ecological functions, and microbial interactions, as well as utilization in industry. Therefore, increased culture efforts combined with culture-independent technologies are required to better understand the uncultured majority.

Culturability varies markedly among environments. For instance, gut microbiomes usually exhibit relatively high cultivation success, with 35 to 65% of molecular species detected by sequencing having representative strains in culture (10). In contrast, extreme environments such as polar regions often show substantially lower success rates. For instance, CPM recovered only 6.37% of bacterial genera detected by metabarcoding in Arctic permafrost soils (11, 12). Polar regions, encompassing the Arctic and Antarctica, are considered unique reservoirs of novel microbial species. Arctic soils, particularly permafrost and active layer soils, are expected to harbor a large pool of psychrophilic and psychrotolerant microorganisms. These microbes are pivotal to climate-relevant biogeochemical cycling (13, 14) and are also promising sources of functional strains and enzymes suited to bioremediation and low-temperature biotechnological applications (15). Accessing the “microbial dark matter” in polar regions is therefore critical for understanding survival mechanisms, metabolic activities, and biogeochemical cycling in the cryosphere, as well as for discovering novel traits of biotechnological value.

However, polar regions are characterized by persistently extreme environmental challenges, including low temperatures, prolonged freezing, freeze-thaw cycles, oligotrophy, desiccation, and strong seasonal UV radiation and light regimes (14, 16, 17), all of which impose severe constraints on microbial activity and growth. Such conditions select for cold-adapted, slow-growing microbial populations that often enter dormant states, maintain low metabolic rates, and exhibit extended lag phases, making them particularly difficult to revive and cultivate under standard laboratory conditions and timescales (18, 19). Consequently, advanced cultivation technologies with higher throughput and efficiency are urgently needed for the isolation and cultivation of microbial species from polar regions.

Cultivation efforts in polar microbiology have revealed new taxa and functions, but the accessible diversity remains limited. Isolation and cultivation of microbes from polar environments usually require long incubation periods (up to several months) at low temperatures (20–22). Traditional plate and liquid enrichment cultures tend to favor relatively fast-growing, copiotrophic lineages, while many rare or strictly cold-adapted groups remain uncultured or underrepresented (22, 23). Previous studies have applied CPM to successfully isolate bacteria from Arctic soils (11, 14, 24–26). Meanwhile, other innovative *in situ* cultivation methods, including cryo-iplate (26), diffusion chambers (12), iTip, and iPore (12), have also been applied to Arctic samples such as lake sediments and soils. These methods recovered different species from plate cultivation but showed limited improvement in cultivation efficiency (26). Despite decades of cultivation efforts, the vast majority of Arctic soil microorganisms remain uncultivated and uncharacterized. Compared with fungi, bacteria showed an even lower rate of culturability in the Arctic soils (11). Therefore, the current gap between the vast uncultured diversity of Arctic soils and the limited cultivation success underscores the need for enhanced methodological throughput.

Microfluidic droplet technology (MDT) has emerged as a transformative platform for microbial cultivation that addresses many limitations of CPM. This technology enables high-throughput encapsulation of single cells into picoliter- to microliter-sized droplets, creating isolated microenvironments that function as miniature bioreactors (27–29). In this way, MDT can reduce interspecific competition by physically isolating individual cells, thereby preventing fast-growing strains from outcompeting slow-growing or dormant cells. Applications of MDT in non-polar soils and gut microbiomes have demonstrated improved cultivation outcomes, including higher richness and recovery of unique genera, compared to CPM (28–30). Moreover, MDT enables the generation of millions of droplets per experiment, significantly increasing the probability of capturing rare and slow-growing taxa. It is particularly suitable for extended incubation periods at low temperatures, as the oil emulsion greatly reduces evaporation as demonstrated previously (31, 32). Collectively, these characteristics suggest that MDT should be suitable for cultivating recalcitrant Arctic soil microorganisms. Nevertheless, to our knowledge, MDT has not yet been applied to Arctic soil microbial isolation and cultivation.

Given the persistent challenges in cultivating Arctic soil microorganisms and the demonstrated capabilities of MDT in accessing previously uncultivated microbial diversity, this study aims to systematically evaluate the performance of droplet-based single-cell isolation and cultivation for Arctic soil microbiota relative to CPM. Through optimization of encapsulation and incubation protocols tailored to cold-adapted, slow-growing bacteria, we aim to establish a high-efficiency pipeline capable of retrieving taxa refractory to standard plate cultivation. This approach is expected to substantially expand the catalog of cultured polar microorganisms, providing critical strain resources to investigate the Arctic “microbial dark matter” and to discover cold-adapted species with biotechnological potential.

## MATERIALS AND METHODS

### Sampling and soil morphology

**Fig 1:**
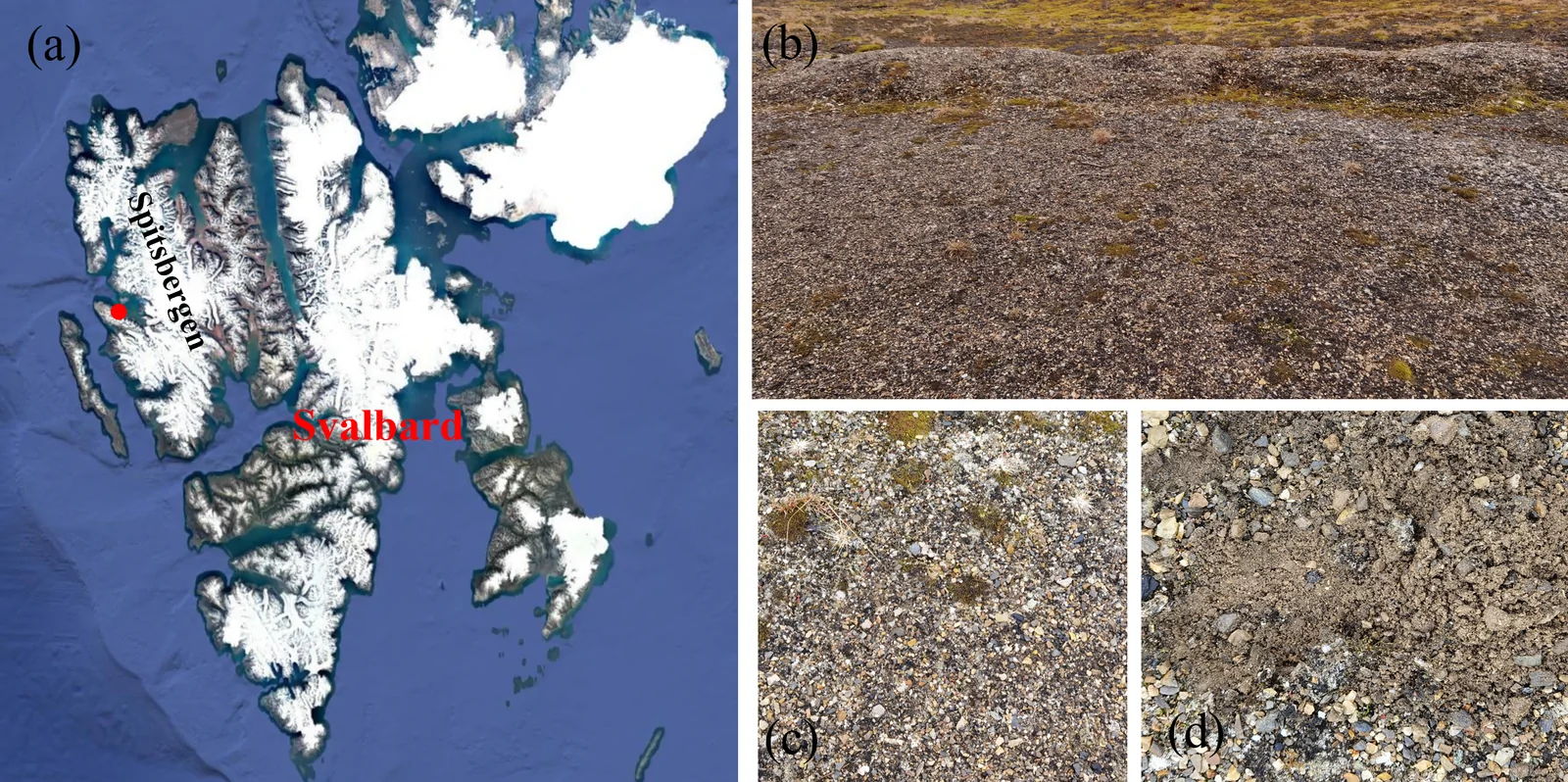
Sampling site information. (**a**) The sampling site, indicated by a red dot, on Spitsbergen Island in Svalbard, in the Norwegian Arctic. Map data: Google, Imagery ©2026 NASA. (**b**) Landscape of the permafrost active layer with sparse vegetation in the background. (**c**) Close-up view of the soil before sampling. (**d**) Soil morphology after removal of the top 1 cm layer using a sterile shovel.

During the Chinese Arctic Scientific Research Expedition at the Yellow River Station in September 2023, active-layer soil samples were collected near Ny-Ålesund on Spitsbergen Island, Svalbard (Fig. 1). This region experiences seasonal freeze–thaw cycles under the influence of climate change, with a mean annual permafrost temperature of –2.5°C (14). The soils in this area are characterized by oligotrophic conditions with low nutrient availability, typical of high Arctic environments (14). The soil exhibited a coarse, gravelly texture, with abundant coarse sand and gravel particles interspersed throughout a fine mineral matrix. The surface layer was poorly developed, with minimal organic accumulation, and comprised a heterogeneous mixture of angular rock fragments and loose mineral soil. The soil structure was weakly aggregated, consistent with recently exposed glacial till or permafrost active-layer material. At the time of sampling, bryophyte-dominated vegetation was visible in the background, whereas the sampling area itself showed minimal bryophyte colonization. The top 1 cm was removed using a sterile shovel, and soil samples were collected into sterile plastic bags and stored at 4°C until transport back to the laboratory in China.

### Cell extraction and pre-evaluation using CPM

Approximately 1.5 g of soil sample was weighed and suspended in a sterile tube containing 15 mL of filtered normal saline (0.9% NaCl). All the following processes were performed in a cold room at 4°C. The soil suspension was subjected to vortex mixing for 5 min and mild sonication (53 kHz) for 1 min in an ultrasonic bath to facilitate cell detachment from soil particles, as described previously (33). Following sonication, the suspension was left to settle at 4°C for approximately 2 h to allow sedimentation of soil debris. The supernatant was diluted 10-fold before cell counting using a hemocytometer under a microscope. The total cell concentration (including both viable and non-viable cells) was estimated by averaging counts from three independent replicate samples. This value was used as the reference for preparing the initial cell suspensions for both MDT and CPM treatments, ensuring identical starting cell numbers within each comparison pair (DropAS_L vs PlateAS_L and DropAS_H vs PlateAS_H). It should be noted that the Poisson distribution model for droplet encapsulation (see below) is based on CFU counts rather than total cell counts. Final serial dilutions of the supernatant at 1:1, 1:10, 1:100, and 1:1000 were prepared for plate colony-forming unit (CFU) counting. To enable direct comparison between CPM and MDT, we used R2A medium (composition: 0.5 g/L yeast extract, 0.5 g/L proteose peptone, 0.5 g/L casamino acids, 0.5 g/L glucose, 0.5 g/L soluble starch, 0.3 g/L sodium pyruvate, 0.3 g/L K₂HPO₄, 0.05 g/L MgSO₄) (34), a formulation commonly used for microbial isolation from polar samples. Aliquots (100 μL) of the serially diluted suspension were spread onto R2A agar plates and incubated at 15°C with daily inspection for 30 days. Plates with approximately 50–100 CFU were used to calculate the viable cell recovery rate, which was approximately 0.04%, substantially lower than the commonly recognized 1%. This value was used as a reference for cell dilution during microdroplet preparation, since no prior culturability data from this sample was available.

### Isolation and cultivation using MDT and CPM

Cell suspension from the above procedure was used for MDT. The single‐cell microliter‐droplet screening system (MISS Cell; TMAXTREE Biotech, Wuxi, China), designed for monoclonal cultivation and picking based on MDT, was employed in this study (32). In the MISS Cell system, the target concentration of the bacterial solution (*c*) was determined based on the λ value in the Poisson distribution model and was defined as CFU/mL rather than total cell count (32). Briefly, the Poisson distribution model assumes that the probability of a droplet containing *x* cells follows *P*(λ, *x*) = λ*ˣ*e⁻^λ^/*x*!, where λ is the theoretical average number of cells per droplet. According to the original description of the MISS Cell system, the recommended λ value ranges from 0.035 to 0.145 to ensure the generation of single-cell droplets (32). Using a λ value of 0.1 and a droplet volume of 2.08 μL (32), the target concentration for encapsulation was calculated as *c* = (1,000/*V*_drop_) × λ = 48 CFU/mL. Since the actual culturability in the microdroplet system was unknown at the time of preparation, we used the culturability ratio obtained from CPM (0.04%) as a rough reference to estimate the corresponding cell density required for microdroplet generation. Although the culturability in the two methods may differ, this approach provided a reasonable starting point for cell dilution in the absence of prior culturability data. Consequently, the cell concentration required for the MISS Cell system was calculated as follows: 48 CFU/mL / 0.04% ≈ 1.2 × 10⁵ cells/mL. A total of approximately 25 mL of this cell suspension was used for droplet generation, yielding approximately 3.0 × 10⁶ cells (labeled DropAS_L). Meanwhile, a 10-fold higher cell input (labeled DropAS_H) was prepared to enhance recovery. Cells were suspended in R2A medium to generate microdroplets using the MISS Cell system at TMAXTREE Biotech (Wuxi, China) (32). The droplet volume was approximately 2.0–2.5 μL, and the cell encapsulation followed Poisson distribution principles to maximize single-cell occupancy. A total of 11,895 and 11,534 droplets were generated for DropAS_L and DropAS_H, respectively. All droplets were spaced by air and stored in coiled transparent Teflon tubes equipped with the MISS Cell system and incubated at 15°C.

Meanwhile, equivalent cell numbers from DropAS_L and DropAS_H were diluted accordingly and spread on R2A agar plates as controls, labeled PlateAS_L and PlateAS_H, respectively. Both DropAS_L and PlateAS_L, starting with the same number of cells, were incubated at 15°C for 30 days, representing the lower-cell-input, longer-incubation regimen. DropAS_H and PlateAS_H, containing 10-fold higher cell numbers, were incubated at 15°C for 17 days, representing the higher-cell-input, shorter-incubation regimen.

### Microdroplet screening and strain picking

At the end of each incubation period, OD_600_ of microdroplets was measured automatically as previously described using the MISS Cell system (32). Microdroplets showing values above the OD_600_ threshold were considered to contain significantly grown cells and were collected into 96-well plates. Up to 100 μL of R2A liquid medium was added to each well to maintain cell viability. The 96-well plates were then stored at 4°C for further downstream analysis. For CPM, CFUs were counted on each agar plate at the end of incubation, and the plates were then stored at 4°C for downstream analysis.

### High-throughput 16S rRNA gene sequencing

To rapidly evaluate cultivation results, 10 μL of cell suspension from each well of the 96-well plates was pooled separately for the DropAS_L and DropAS_H regimens. Notably, each well was mixed by pipetting before sampling to avoid cell sedimentation. The pooled cells were centrifuged to remove liquid medium and stored at −80°C before sequencing. Meanwhile, all colonies grown on agar plates were collected using an inoculation loop with an appropriate volume of sterile 0.9% NaCl solution and transferred to tubes. Liquid was removed by centrifugation before freezing at −80°C.

DNA was extracted using the MagBeads FastDNA Kit for Soil (MP Biomedicals, CA, USA) for all samples. To improve the resolution of species identification, we used 27F (5′–AGAGTTTGATCCTGGCTCAG–3′) and 1492R (5′–GGTTACCTTGTTACGACTT–3′) primers to amplify the near-full-length 16S rRNA gene and sequenced the amplicons using a PacBio Sequel platform at Shanghai Personalbio Technology CO., LTD. (Shanghai, China).

### PacBio sequence analysis

Raw data from PacBio Sequel platform were processed using CCS v4.0.0 software (--min-length 500, --max-length 3000, with default parameters for all other parameters) (https://github.com/PacificBiosciences/ccs) (35), generating highly accurate single-molecule consensus reads with base quality values. FASTQ files were demultiplexed based on barcode sequences at both ends of the reads. Barcodes were removed, and reverse-complement sequences were converted to the forward orientation according to the primer sequences.

Near-full-length PacBio sequences were processed using the QIIME 2 pipeline (version 2024.2.0) (36). In brief, sequences were imported into QIIME 2 as single-end reads and processed through the following steps: (i) trimming and quality filtering using the cutadapt trim-single plugin with a minimum length threshold of 1,200 bp (--p-minimum-length 1200); (ii) dereplication of trimmed sequences using the vsearch dereplicate-sequences plugin to collapse identical sequences; and (iii) chimera detection and removal using the vsearch uchime-denovo plugin. The resulting high-quality, non-chimeric sequences were used for downstream analysis. Taxonomic classification was performed using the feature-classifier classify-sklearn plugin against the SILVA reference database (SILVA v138.1, full-length 16S rRNA genes).

### Comparison with type strains

To investigate the potential novelty of isolates retrieved from both CPM and MDT, a database of 16S rRNA gene sequences from type strains was constructed. In brief, a list of all available type strains at the time of this work (15 January 2026) was downloaded from the List of Prokaryotic Names with Standing in Nomenclature (LPSN) (https://lpsn.dsmz.de/downloads), an authoritative online database providing information on type strains validly published according to the International Code of Nomenclature of Prokaryotes (ICNP) (37). Meanwhile, all 16S rRNA gene sequences were downloaded from the NCBI database. In-house Python scripts were used to extract 16S rRNA sequences according to the LPSN list and to filter matched sequences containing six consecutive N’s to retain high-quality sequences for downstream analysis. The resulting type strain reference database (retaining 19,958 sequences between 600 and 1,600 bp) was used for BLASTn comparison. High-quality, non-chimeric near-full-length 16S rRNA gene sequences from this study were compared against the type strain database using BLASTn with parameters -evalue 1e-5 and -max_target_seqs 1. The 16S rRNA genes of potential novel taxa were aligned using MAFFT v7.520 (38). Phylogenetic trees were reconstructed using IQ-TREE version 2.2.6 with 1,000 ultrafast bootstrap replicates (-bb 1000) and the best-fitting substitution model determined automatically by the ModelFinder Plus (-m MFP) (39). Phylogenetic trees were annotated using the interactive iTOL online platform (40).

### Strain identification and verification

To verify the sequencing results, microdroplets were randomly selected and further purified on R2A agar plates. Colonies with distinct morphologies were chosen for genomic DNA extraction and 16S rRNA gene amplification using primers 27F and 1492R for Sanger sequencing. Sequences were identified by submitting to the EzBioCloud server (https://www.ezbiocloud.net) (41).

## RESULTS

### MDT achieved higher recovery than CPM

**Fig 2:**
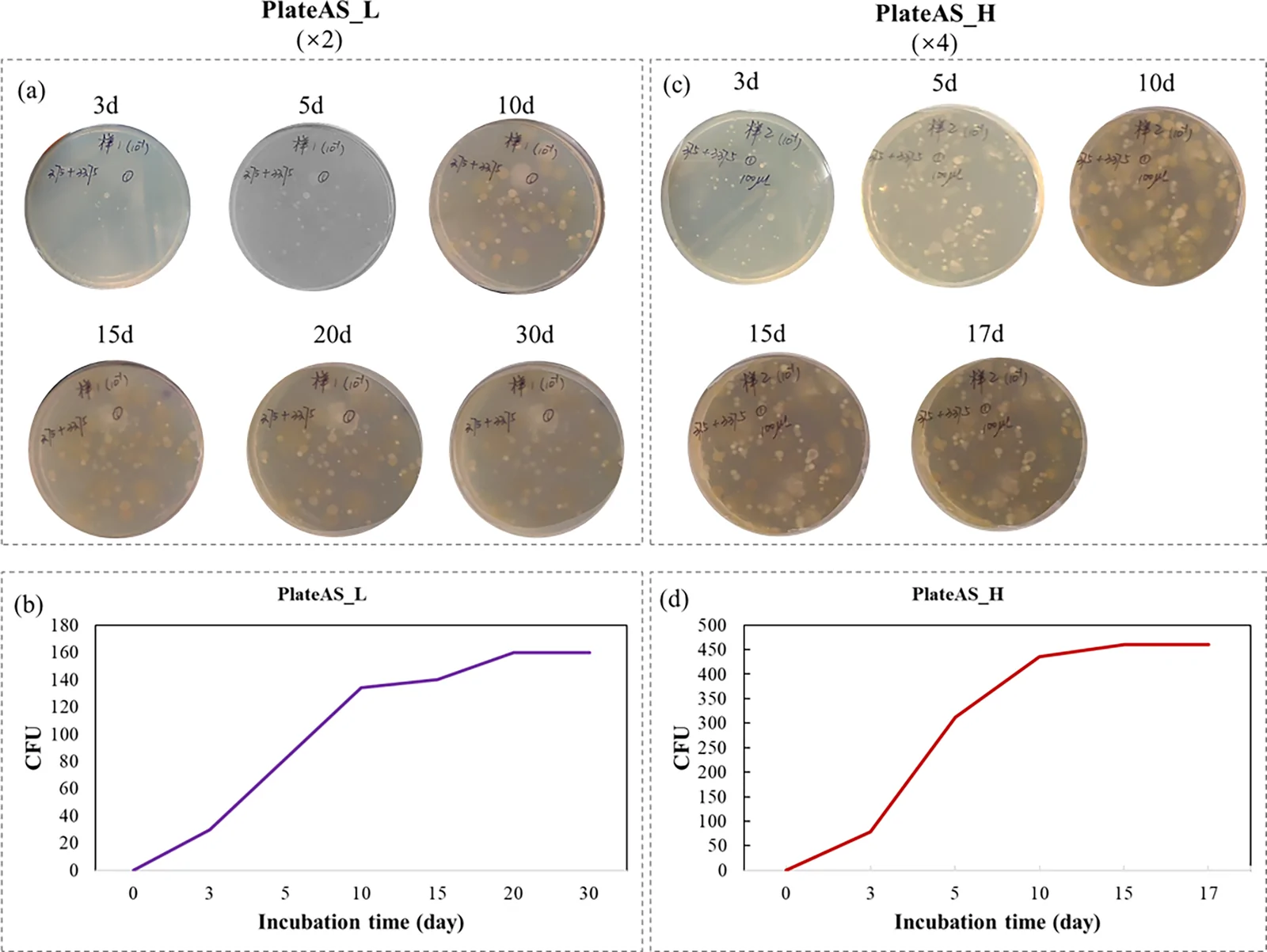
Standard plate-based method results. (**a**) Cells equivalent to those used in DropAS_L were equally spread onto two plates; one representative plate is shown. (**b**) Total CFUs on the two plates in panel a were counted manually at various incubation times. (**c**) Cells equivalent to those used in DropAS_H were equally spread onto four plates; one representative plate is shown. (**d**) Total CFUs on the four plates in panel c were counted manually at various incubation times.

The results demonstrated a dramatic improvement in isolate recovery with MDT. Specifically, the PlateAS_L group (15°C, 30 days) yielded approximately 160 visible CFU by the end of incubation, whereas the PlateAS_H group (15°C, 17 days) produced approximately 450 CFU (Fig. 2). Notably, the PlateAS_L group exhibited substantial fungal contamination, likely due to the prolonged 30-day incubation, although the possibility of slow-growing indigenous fungi from the soil could not be completely excluded. In contrast, the DropAS_L group (15°C, 30 days) yielded up to 1,300 microdroplets with visible growth, while the DropAS_H group (15°C, 17 days) yielded 2,939 microdroplets. Given that both CPM and MDT started with the same number of input cells per comparison, the recovery rate of MDT was calculated to be 8.1-fold (1,300/160) and 6.5-fold (2,939/450) higher than that of CPM, respectively.

### MDT improved isolation throughput and efficiency

**Fig 3:**
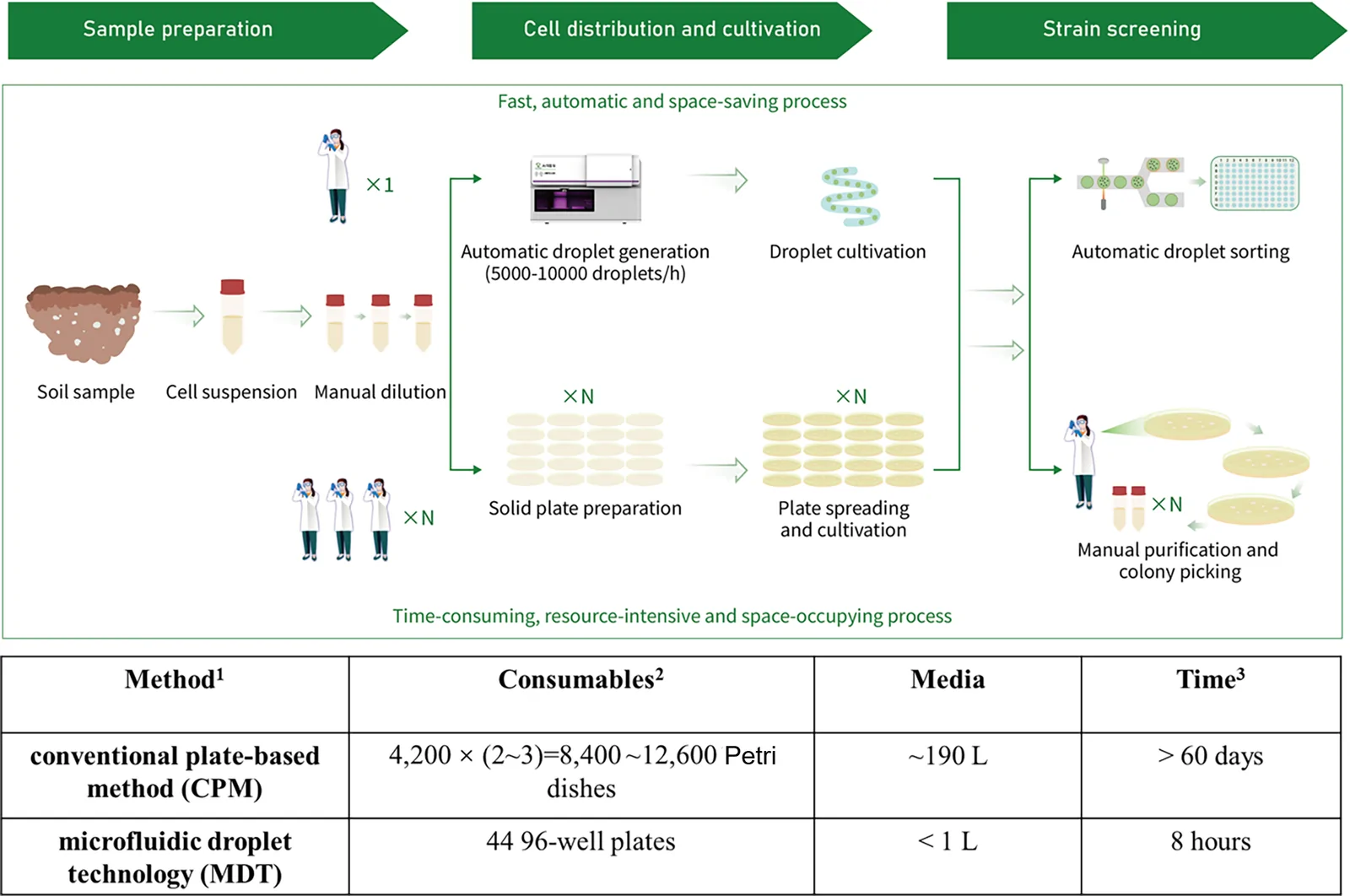
Comparison of two different approaches for isolation, cultivation, and screening: conventional plate-based method (CPM) and high-throughput microfluidic droplet technology (MDT). (1) The calculation is based on the recovery numbers of colonies from plates and of microdroplets achieved in this study, assuming a target of obtaining 4,200 strains using either method. (2) The standard plate culture technique typically requires two to three rounds of streak purification to obtain a pure culture. Only the main consumables are included for comparison. (3) Based on a single person completing 200 plate spreading/streaking operations per day, the minimum time required for plate preparation and subsequent spreading/streaking was calculated. This result should not be treated as an absolute value but rather as a theoretical estimate.

MDT substantially outperformed CPM in both efficiency and throughput. Cell dispersion and isolation were fully automated using the MISS Cell system, operating at a throughput of 5,000–10,000 droplets per hour, which is considerably more efficient than manual spreading and colony picking on agar plates (Fig. 3). In this study, following MDT processing of two batches, up to 4,239 microdroplets containing isolates that exhibited significant growth above the threshold designated by the MISS Cell system were collected. We assume that a single researcher works an 8-h day and can manually prepare, coat, and streak a maximum of 200 agar plates per day, which is generally consistent with standard laboratory practice. To obtain pure cultures of 4,200 isolates by the conventional plate method, each candidate colony would require two to three rounds of streak purification, totaling approximately 8,400 to 12,600 plates. At 200 plates per day, this translates to 42 to 63 working days. Including additional time for medium preparation, sterilization, and routine observation (approximately 10–15 days), the total hands-on time would be approximately 60 to 75 working days. In contrast, MDT accomplished the same task in approximately 8 h of instrument runtime, representing a more than 180-fold improvement in efficiency (Fig. 3). However, this is a theoretical estimate based on the above assumptions, and further droplet downstream processing, such as validation and purification, is not included in this calculation, although in theory, most droplets should contain only a single colony. Nevertheless, MDT substantially surpassed CPM in overall efficiency. Furthermore, MDT consumed substantially fewer consumables, reagents, and medium than CPM and required considerably less space for incubation and other associated processes.

### MDT recovered higher taxonomic richness and evenness at all levels

To enable comparison of species composition recovered by MDT and CPM at higher phylogenetic resolution, over 70,000 near-full-length 16S rRNA gene sequences were generated per experiment using the PacBio Sequel platform. After filtering, trimming, dereplication, and chimera removal using the QIIME 2 pipeline, 23,423–39,899 final amplicon sequence variants (ASVs) longer than 1,200 bp were retained for downstream analysis (Table 1). Within both MDT and CPM, high-cell-input groups (DropAS_H and PlateAS_H) yielded a greater number of ASVs than corresponding low-cell-input groups (DropAS_L and PlateAS_L). Sequences were annotated at various taxonomic levels from phylum to genus (Table 1). Across all samples, a total of 22 phyla and 283 genera were identified after excluding unclassified taxa. *Pseudomonadota*, *Bacteroidota*, *Actinomycetota*, and *Bacillota* were the dominant phyla. At all taxonomic levels, DropAS_H always ranked first among them. Overall, MDT groups contained a higher number of taxa than CPM controls across all taxonomic levels. Specifically, DropAS_H contained 256 genera (including unclassified genera), 45 more than 211 detected in PlateAS_H. Similarly, DropAS_L yielded 59 more genera than PlateAS_L. At the phylum level, three and two additional phyla were detected in DropAS_H and DropAS_L, respectively, compared to PlateAS_H and PlateAS_L.

**TABLE 1:** Sequence quality control and taxonomic annotation based on PacBio sequencing of pooled samples (ASVs)

Experiment design	Sequence quality control			Taxonomic annotation				
	Raw reads	Filtered reads	Clean ASVs (>1,200 bp)	Genus	Family	Order	Class	Phylum
DropAS_H	75,127	58,573	39,899	256	122	67	31	19
PlateAS_H	73,967	57,619	36,854	211	107	57	25	16
DropAS_L	73,821	61,292	23,423	138	63	38	15	10
PlateAS_L	73,755	61,120	24,637	79	48	30	12	8

### MDT enhanced recovery of rare genera and reduced copiotroph abundance

**Fig 4:**
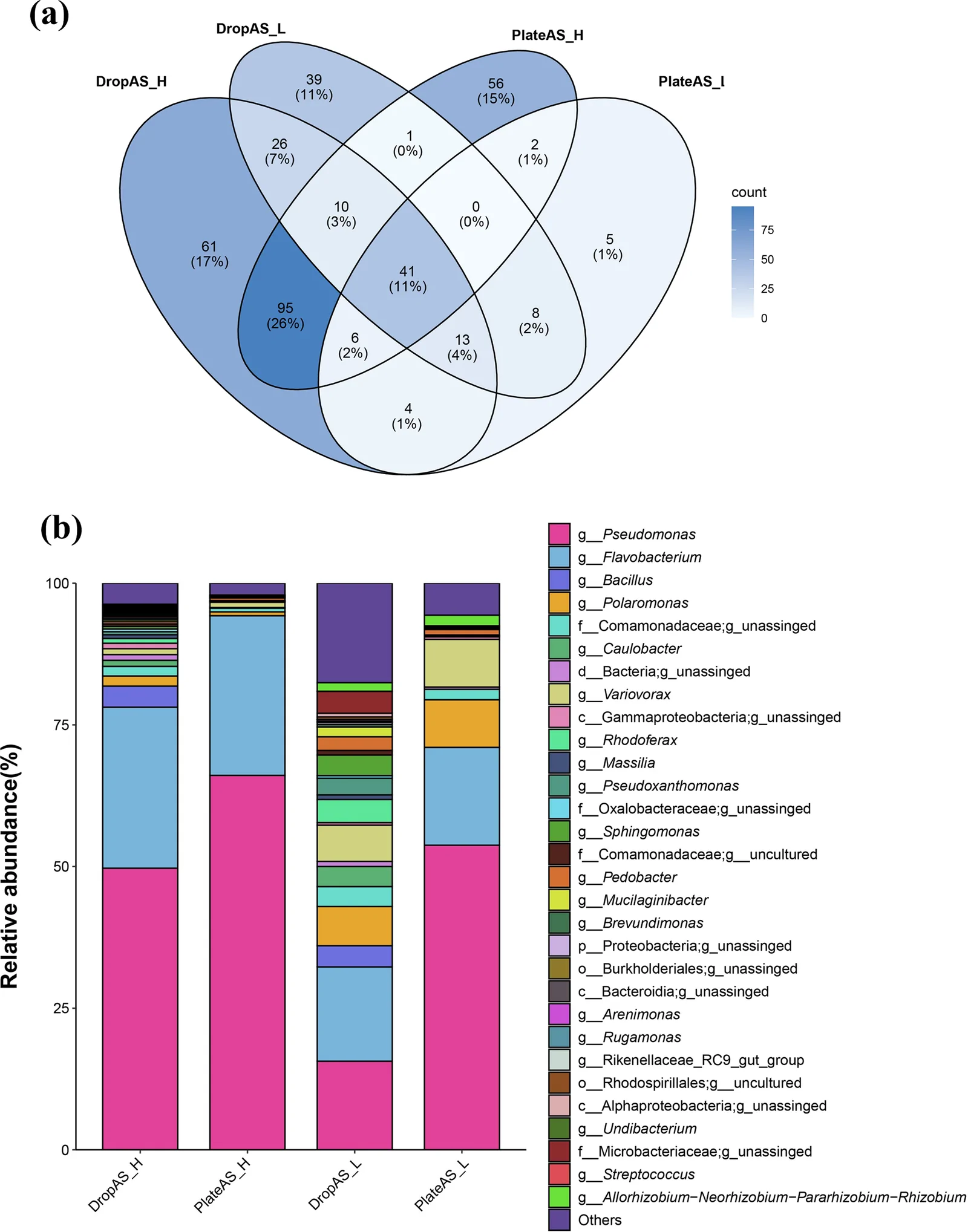
Comparison of genera detected across isolation experiments. (**a**) Venn diagram of shared genera. (**b**) Composition and distribution of top 30 genera.

To focus on the comparison between MDT and CPM, genera recovered across all four groups were analyzed in detail. The number of genera detected varied considerably, with only 41 genera (11%) shared among the four groups (Fig. 4a). The highest number of unique genera was detected in DropAS_H (61, 17%), whereas the lowest was in PlateAS_L (5, 1%), representing a 12.2-fold increase. The second highest number of unique genera was detected in PlateAS_H (56, 15%), followed by DropAS_L (39, 11%).

Although two genera, *Pseudomonas* and *Flavobacterium*, dominated in all four experiments, their relative abundances varied markedly (Fig. 4b). Notably, *Pseudomonas* and *Flavobacterium* accounted for up to 66.1% and 28.2% of reads in PlateAS_H (the highest), but only 15.6% and 16.6% in DropAS_L (the lowest), respectively. Another apparent difference between MDT and CPM groups was the evenness of genus distribution. MDT groups exhibited both higher genus richness and greater evenness than corresponding CPM controls. Moreover, DropAS_L contained the highest percentage of rare genera (i.e., those outside the top 30 shown in Fig. 4b) (17.5% in DropAS_L vs 2.1% in PlateAS_H).

### MDT and CPM enriched different groups

**Fig 5:**
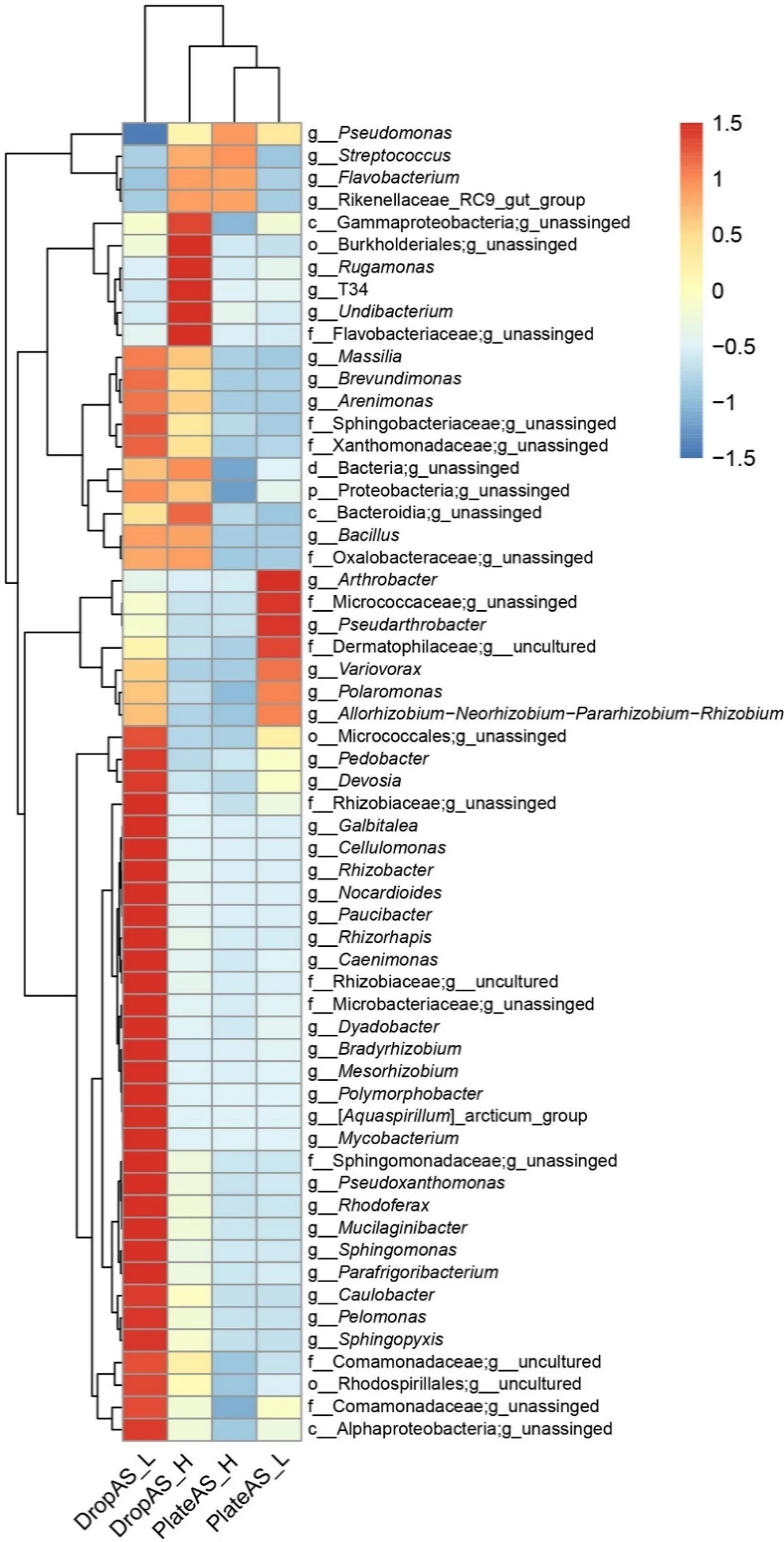
Heatmap of genera with abundance over 0.1%.

Different taxa were enriched between MDT (DropAS_L and DropAS_H) and CPM groups (PlateAS_L and PlateAS_H) (Fig. 5). Specifically, unclassified bacteria at various taxonomic levels, from domain to family, including *Bacteriodia*, *Pseudomonadota*, *Oxalobacteraceae*, *Xanthomonadaceae*, and *Sphingobacteriaceae*, were much more abundant in MDT (up to >40-fold higher) than in CPM groups. Besides, genera *Bacillus*, *Massilia*, *Mucilaginibacter*, *Brevundimonas*, and *Arenimonas* were most abundant in the MDT group (up to >1,600-fold higher). Moreover, genera with relative abundances >0.01%, including *Caulobacter* (1.1%, 3.6%), unclassified *Caulobacteraceae* (0.05%, 0.09%), *Sphingomonas* (0.5%, 3.6%), *Arenimonas* (0.2%, 0.3%), *Cryobacterium* (0.06%, 0.06%), *Pelomonas* (0.06%, 0.3%), *Nocardioides* (0.04%, 0.9%), *Paucibacter* (0.03%, 0.7%), *Sphingopyxis* (0.03%, 0.1%), *Rhizorhapis* (0.03%, 0.3%), *Uliginosibacterium* (0.02%, 0.03%), *Sphingobium* (0.02%, 0.04%), and unclassified *Cellulomonadaceae* (0.01%, 0.02%), were exclusively detected in the MDT (values in parentheses represent relative abundances in DropAS_H and DropAS_L, respectively) but absent in CPM.

Specific taxa were enriched in DropAS_L, including genera *Pedobacter*, *Devosia*, *Galbitalea*, *Cellulomonas*, *Rhizobacter*, *Nocardioides*, *Paucibacter*, *Rhizorhapis*, *Caenimonas*, *Dyadobacter*, *Bradyrhizobium*, *Mesorhizobium*, *Polymorphobacter*, *Mycobacterium*, *Pseudoxanthomonas*, *Rhodoferax*, *Mucilaginibacter*, *Sphingomonas*, *Parafrigoribacterium*, *Caulobacter*, *Pelomonas,* and *Sphingopyxis*, as well as unclassified groups from *Alphaproteobacteria*, *Micrococcales*, *Rhizobiaceae*, *Microbacteriaceae*, *Sphingomonadaceae*, *Comamonadaceae*, and *Rhodospirillales* (Fig. 5).

Although the most apparent difference existed between MDT and CPM groups, long incubation tended to enrich specific groups in both DropAS_L and PlateAS_L, including *Pseudarthrobacter*, *Allorhizobium-Neorhizobium-Pararhizobium-Rhizobium*, *Polaromonas*, and *Variovorax*. In particular, genera *Arthrobacter* and *Pseudarthrobacter*, as well as unassigned genera in *Micrococcaceae* and *Dermatophilaceae*, were highly enriched in PlateAS_L.

### MDT recovered more potential novel taxa

To identify potential novel species and genera recovered in the four experiments, 16S rRNA gene sequences were retrieved for validly published type strains available in the LPSN database and used as the database for BLASTn (see Materials and Methods). According to the widely used 98.65% and 95% 16S rRNA identity thresholds for novel species and genera (8), 49.5% and 27.2% of sequences that matched type strains in DropAS_H represented, respectively, potential novel species and genera, which were the highest among all experiments (Table 2). In contrast, the lowest percentages of potential novel genera (22.2%) and species (9.8%) were detected in PlateAS_H. Overall, about 1.6- to 2.2-fold more potential novel species and genera were detected in MDT groups than in CPM groups.

**TABLE 2:** Statistics of potential novel taxa based on PacBio sequencing of pooled samples (ASVs)

Experiment design	Clean ASVs	Number of ASVs belonging to potential novel taxa (based on 16S rRNA gene identity)			
		Species (<98.65%)	Genus (<95%)	Family (<86.5%)	Order (<82%)
DropAS_H	39,899	19,756 (49.5%)	10,864 (27.2%)	436 (1.1%)	27 (0.07%)
PlateAS_H	36,854	8,197 (22.2%)	3,615 (9.8%)	246 (0.67%)	30 (0.08%)
DropAS_L	23,423	11,181 (47.7%)	3,532 (15.1%)	103 (0.44%)	1 (0.004%)
PlateAS_L	24,637	7,271 (29.5%)	2,953 (12.0%)	36 (0.15%)	0 (0%)

**Fig 6:**
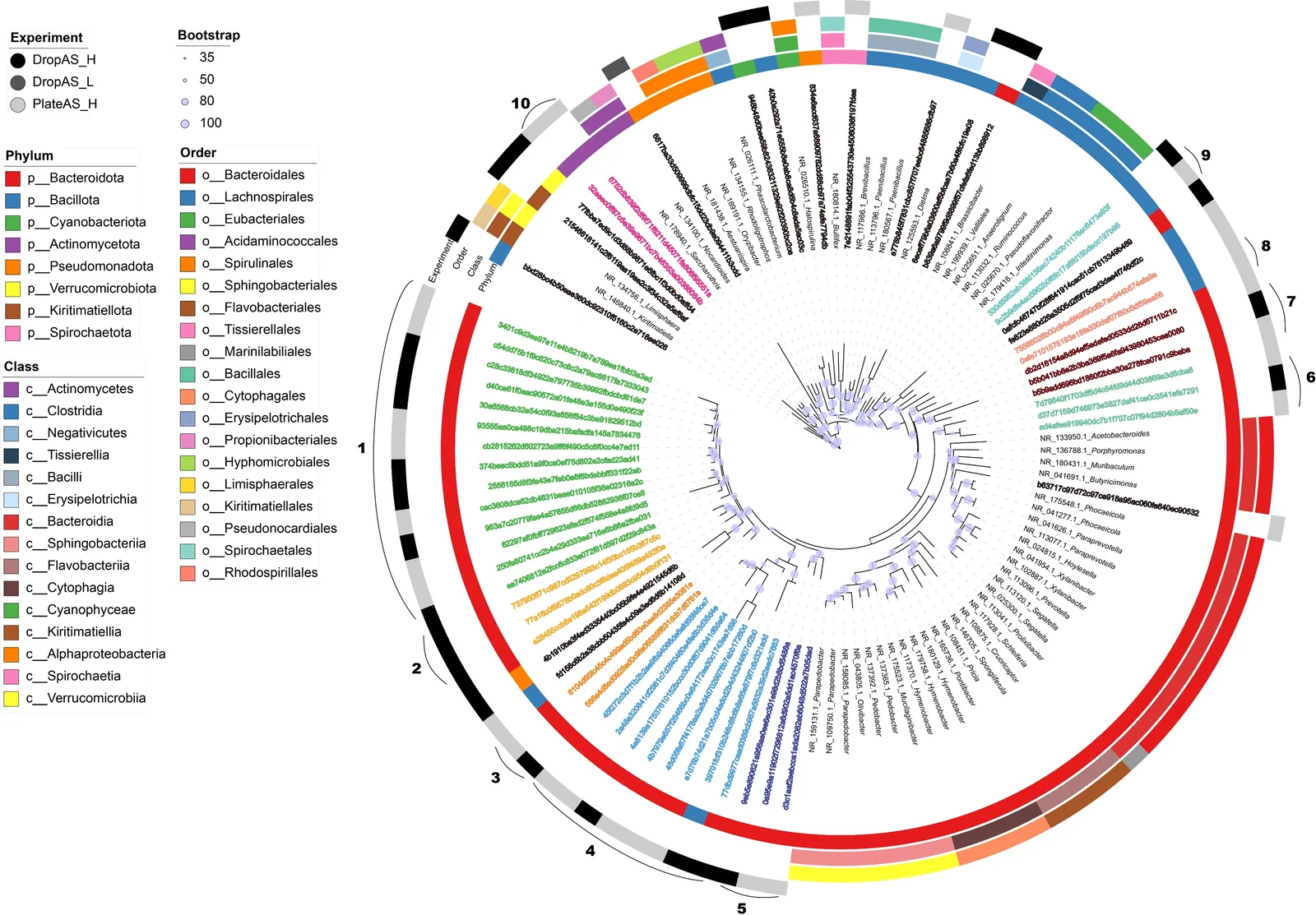
Phylogenetic tree of sequences sharing less than 82% identity with type strains. Four concentric rings (from inner to outer) denote phylum, class, order, and experimental groupings, respectively. Sequences from this study are identified by their Feature IDs, while type strains are labeled with accession numbers and genus names. Leaf node colors reflect different putative clusters, which correspond to the numeric labels and curved lines above the outermost ring (marked “Experiment”). Bootstrap support values are represented as circles on branches, with the size scale explained in the legend on the left. The color key is also provided on the left.

To further investigate the novelty of sequences recovered from this study, sequences sharing less than 82% identity with type strains, representing potential novel orders (8), were pooled to construct a phylogenetic tree with their closest type strains (Fig. 6). These included 27 sequences from DropAS_H (belonging to six phyla: *Bacillota*, *Bacteroidota*, *Pseudomonadota*, *Kiritimatiellota*, *Cyanobacteriota*, and *Verrucomicrobiota*), 30 from PlateAS_H (belonging to five phyla: *Bacillota*, *Bacteroidota*, *Pseudomonadota*, *Spirochaetota*, and *Actinomycetota*), and one from DropAS_L belonging to *Pseudomonadota*. More than half of the sequences recovered from this study clustered separately from type strains. In particular, 10 potential clusters were identified as putative novel orders that were not closely related to known groups, most of which belonged to *Bacteroidota*. In addition to these 10 potential novel clusters, other sequences from this study may represent novel taxa above the family level from rare phyla such as *Spirochaetota*, *Bacillota*, *Kiritimatiellota,* and *Verrucomicrobiota*.

### Strain isolation verification

Fifty microdroplets with varying OD_600_ values, representative of different growth rates, were randomly selected for validation, given the labor-intensive nature of downstream processing and the need for preliminary confirmation of cultivation success. Generally, uniform colonies appeared on R2A plates derived from individual microdroplets, indicating that each droplet likely contained a single cultivated species. A total of 20 colonies with distinct morphologies were chosen for Sanger sequencing. Finally, 17 strains were successfully sequenced (14 in both directions, three in only one direction), whereas three failed during DNA extraction and sequencing (Table 3). These 17 strains showed 16S rRNA gene sequence identities ranging from 92.92% to 100% with their closest type strains. For the three partially sequenced strains, the identities should be considered preliminary; full-length sequencing would be required for precise species-level assignment. Among them, 12 out of 17 showed identities below 98.65%, representing potential novel species within *Arthrobacter*, *Bacillus*, *Flavobacterium*, *Mesorhizobium*, *Nocardioides*, *Polaromonas*, and *Sphingomonas*. One strain, labeled S-114-7H, showed <95% identity with the closest type strain *Devosia insulae* DS-56, suggesting a potentially novel genus within the family *Devosiaceae* of the phylum *Pseudomonadota*. All genera obtained here were also detected in the high-throughput PacBio sequencing results, with C*ellulomonas*, *Devosia*, *Nocardioides*, *Mesorhizobium*, *Sphingomonas*, *Polaromonas*, and *Bacillus* enriched in MDT experiments (Fig. 6). These results confirm the reliability of MDT sequencing data and demonstrate the feasibility of MDT for obtaining novel species from Arctic soil samples.

**TABLE 3:** Identification of pure strains isolated from microdroplets and confirmed by Sanger sequencing

Strain	Closest type strain	Identity	Coverage	Phylum
S-133-4B	C*ellulomonas humilata* ATCC 25174	99.31%	60.60%^*a*^	*Actinomycetota*
*S-132-11H* ^*b*^	*Mesorhizobium norvegicum* 10.2.2 *Mesorhizobium loti* NBRC 14779	97.86%	99.00%	*Pseudomonadota*
S-132-10C	*Nocardioides allogilvus* CFH 30205	98.37%	55.40%^*a*^	*Actinomycetota*
S-114-7H	*Devosia insulae* DS-56	92.92%	98.00%	*Pseudomonadota*
S-114-7G	*Sphingomonas silvisoli* RP18	96.27%	98.00%	*Pseudomonadota*
S-114-7F	*Pelomonas dachongensis* DC23W	99.06%	96.00%	*Pseudomonadota*
S-114-7E	*Mesorhizobium norvegicum* 10.2.2	99.00%	98.00%	*Pseudomonadota*
*S-112-9D* ^*b*^	*Mesorhizobium norvegicum* 10.2.2 *Mesorhizobium loti* NBRC 14779	97.14%	97.00%	*Pseudomonadota*
S-112-1B	*Polaromonas jejuensis* NBRC 106434	95.74%	98.00%	*Pseudomonadota*
S-101-1G	*Polaromonasginsengisoli* Gsoil 115	97.19%	99.00%	*Pseudomonadota*
F-143-8A	*Pseudomonas yamanorum* 8H1	100%	54.60%^*a*^	*Pseudomonadota*
F-143-3D	*Flavobacterium collinsii* 983-08	96.70%	98.00%	*Bacteroidota*
F-141-G11	*Arthrobacter ginsengisoli* DCY81	96.85%	98.00%	*Actinomycetota*
F-141-B9	*Bacillus cereus* ATCC 14579	97.73%	99.00%	*Bacillota*
F-141-A9	*Bacillus cereus* ATCC 14579	98.17%	98.00%	*Bacillota*
F-114-12D	*Polaromonas ginsengisoli* Gsoil 115	98.52%	98.00%	*Pseudomonadota*
F-113-G9	*Bacillus cereus* ATCC 14579	98.02%	97.00%	*Bacillota*

^*a*^ These three strains were sequenced from one direction only. Hence, the coverage is lower than the others.

^*b*^ Strains S-112-9D and S-132-11H showed identical 16S rRNA gene sequence identities to two different type strains. This likely reflects the high sequence similarity between the two reference species themselves.

## DISCUSSION

This study represents the first application and systematic evaluation of MDT for isolation and cultivation of microorganisms from Arctic active-layer soils. Our results demonstrate that MDT not only increased isolation throughput by more than 180-fold compared to CPM but also, more importantly, substantially improved the representativeness of recovered community composition. Under identical starting cell numbers and R2A medium conditions, MDT recovered 21–75% more genera than CPM, with a significantly more even community distribution. For instance, the combined relative abundance of dominant copiotrophic genera *Pseudomonas* and *Flavobacterium* decreased from 94.3% in CPM to 32.2% in MDT. These findings align with previous MDT applications in gut microbiomes and non-extreme soils, where droplet confinement is thought to alleviate interspecific competition and release slow-growing or rare taxa that are otherwise outcompeted by fast-growing strains (29, 42). This interpretation is consistent with our observations, specifically, the reduced relative abundance of copiotrophs and increased recovery of rare genera in MDT compared to CPM. However, we acknowledge that our current data cannot definitively distinguish whether this shift results from alleviated competition, physical isolation *per se*, or simply from the dilution of copiotroph reads by the successful cultivation of additional taxa. While previous studies have provided evidence for competition alleviation in droplet systems (29, 42), targeted strain interaction analyses (e.g., pairwise co-culture experiments) will be required to confirm the underlying mechanisms in our system. Overall, high-throughput methods such as MDT can effectively address the limitations of CPM, particularly for extreme environment samples that harbor slow-growing microbes and require prolonged incubation. Beyond these performance gains, the scalability and efficiency of MDT offer distinct advantages for large-scale investigations. As a powerful complement to meta-omics approaches, it enables the high-throughput construction of microbial strain collections from polar regions. As demonstrated in this study and elsewhere (27, 43), MDT dramatically reduces labor input, increases throughput, and conserves reagents and consumables, thereby providing a practical and robust pipeline for accessing the untapped cultural diversity of Earth’s cryosphere. Beyond these global community-level improvements, MDT also enabled the recovery of specific taxa that are ecologically or biotechnologically significant, as illustrated below.

Among the MDT-enriched genera, *Caulobacter* is particularly noteworthy. This genus is a classic model for studying asymmetric cell division and polar development, characterized by a distinctive stalk with a holdfast adhesin at its distal end that attaches to solid-liquid interfaces (44). Although *Caulobacter* has long been regarded primarily as an aquatic oligotroph, recent metagenomic analyses challenge this view, demonstrating that its relative abundance in soils is on average fourfold higher than in aquatic environments (45). The successful recovery of *Caulobacter* from Arctic soil in this study supports this revised distribution pattern and further suggests its active adaptation to low-nutrient conditions and periodic freeze-thaw cycles at solid-liquid interfaces. Interestingly, *Caulobacter* was specifically enriched in MDT (1.1% in DropAS_H and 3.6% in DropAS_L) yet completely absent in CPM. One plausible explanation relates to the contrasting physical environments: CPM provides only a solid agar surface, whereas MDT generates a liquid–solid–air interface. This hypothesis is supported by a previous report that *Caulobacter* also colonizes the air–liquid interface (46). Further investigation is needed to fully elucidate the basis for this differential recovery. Nevertheless, this differential recovery underscores the superior capacity of MDT over CPM in accessing specific oligotrophic-adapted bacterial groups from complex environmental samples.

Beyond *Caulobacter*, several genera enriched or exclusively detected in MDT possess clear biotechnological applications and value for studying adaptation to the Arctic soil environment. For instance, the genus *Bacillus*, which was entirely absent in PlateAS_H but reached 3.7% in MDT, serves as a major chassis (in particular *B. subtilis*) for industrial protein expression and as plant growth-promoting microbial fertilizers (47). Cold-adapted *Bacillus* strains from Arctic soils could be exploited as platforms for low-temperature enzyme expression or as biofertilizers for cold-region agriculture. The genus *Massilia* was enriched over 1,600-fold in MDT relative to CPM. Multiple novel *Massilia* species have been described from Antarctic streams, lakes, and regoliths, demonstrating strong adaptation to extreme cold and oligotrophic conditions (48, 49). Genera such as *Cryobacterium*, *Mucilaginibacter*, and *Arenimonas* are well-known cold-adapted bacteria widely distributed in cryoconite, glacial ice, and permafrost soils, representing characteristic members of indigenous cryospheric microbial communities (50). Additionally, *Paucibacter* (known for microcystin and nodularin detoxification) (51) and *Sphingopyxis* (capable of pesticide degradation) (52) were enriched in MDT, suggesting potential applications in cryosphere bioremediation. Genera such as *Nocardioides*, *Massilia*, and *Mucilaginibacter* are also recognized for active secondary metabolite production, with potential biomedical value (53). Altogether, the microbial cultures obtained in this study, especially from MDT, showed great potential in understanding mechanisms behind adaptation to cold and nutrient depletion and also in future applications in various fields.

Our study differs from previous MDT applications in several key aspects. First, we used a larger volume (2.0–2.5 μL/droplet) rather than picoliter volumes commonly employed in previous work. Although this reduces the total number of droplets generated, it substantially facilitates downstream processing, including long-term incubation stability, single-droplet dispensing into microwells, and preservation of droplets for subsequent validation and strain purification. This volume trade-off is particularly suitable for slow-growing polar microorganisms that require extended incubation. Second, we utilized near-full-length 16S rRNA gene sequencing via PacBio for the first time in MDT cultivation assessment. This approach provides substantially greater phylogenetic resolution and higher confidence in species identification. Previous MDT studies evaluating cultivation outcomes relied almost exclusively on short-read sequencing of partial 16S rRNA gene fragments (~250–300 bp, typically the V4 region) (28, 29), which provides limited resolution for species-level identification and novel taxon delineation. Third, our study is the first to evaluate MDT applicability for extreme environmental samples from the cryosphere. Unlike prior MDT studies conducted at 30–37°C with short incubation (typically 1–5 days) (29, 42), we optimized the cultivation temperature to 15°C and extended the incubation period to 30 days. This regimen was necessary to accommodate the slow growth rates and extended lag phases characteristic of cold-adapted soil microbiota. The water-in-oil emulsion system, combined with sealed Teflon tubing incubation, effectively prevented both evaporation and airborne contamination, issues that frequently compromise long-term incubation using CPM. Previous MISS Cell data demonstrated that this oil-sealed system reduced 5-day methanol evaporation to only 12.7%, compared to 86.7% in deep-well plates and 55.9% in shake flasks (31, 32). Given that aqueous liquid media are expected to be retained even longer than methanol, which evaporates more rapidly, the protective effect of this closed system is anticipated to be even more pronounced for water-based culture systems. In addition, the closed Teflon tube system physically isolates the droplets from external airborne contaminants, as shown previously (32, 54). Thus, our results demonstrate that MDT is particularly suitable for environmental samples from the cryosphere, offering a robust platform for long-term, low-temperature incubation without the desiccation and fungal contamination problems commonly encountered in CPM.

Despite the demonstrated advantages of MDT, this study has several limitations that point to future improvements. First, we only used one cultivation medium, R2A, under aerobic conditions in this study. Although R2A medium is a standard oligotrophic formulation widely used for microbial isolation from the polar regions (24, 55) and was appropriately chosen here to evaluate the baseline applicability of MDT in Arctic soils, its use alone inevitably excluded chemolithoautotrophs, strict anaerobes, and taxa requiring specific substrates for growth. Media supplemented by soil extract were shown to increase the cultivation diversity in a previous MDT study (28). Future efforts should therefore expand the cultivation repertoire to include multiple media, as well as variations in oxygen levels and temperature, to capture the full physiological diversity of the cryosphere microbiome, as proposed previously (12). In addition, biological replicates were not included in the current experimental design. As this study represents an initial methodological evaluation of MDT to Arctic soils, the primary objective was to establish a baseline comparison between MDT and CPM under controlled conditions rather than to comprehensively optimize all parameters. We acknowledge that the inclusion of biological replicates would further enhance the statistical robustness of the findings, and this will be an essential component of future studies aimed at systematically optimizing MDT protocols for polar and other extreme environments. Another constraint lies in the downstream recovery of novel taxa. Although PacBio sequencing revealed substantial proportions of potential novel species (49.5% in DropAS_H) and novel genera (27.2%), the transition from sequencing-based detection to physical isolation of higher-order novel taxa (e.g., novel families or orders) remains challenging. It should be noted that the potential novel taxa recovered in this study are determined based on 16S rRNA genes, and these novelty estimates should be interpreted with caution because mixed populations within droplets may exist. Specifically, the cell suspension inevitably contains viable but non-culturable (VBNC) cells that are not detected by plate counting. This may cause the actual encapsulation rate to deviate from Poisson predictions and lead to a higher proportion of double- or multiple-occupancy droplets than expected. Such mixed populations could compromise clonal purity and introduce uncertainty into novelty estimates based on pooled droplet sequencing. Therefore, whole-genome sequencing together with comprehensive polyphasic taxonomic characterization will be essential to formally establish the taxonomic status of these isolates. The targetability of downstream analysis is currently limited due to the need to screen thousands of droplets to locate a target strain present as a low-abundance sequence in the pooled library. Future integration of in-droplet identification technologies could bridge this gap by enabling targeted recovery of specific taxa directly from the droplet pool. One promising approach is barcoded pooled sequencing, in which each droplet is co-encapsulated with a uniquely barcoded bead, followed by cell lysis and either 16S rRNA gene amplicon PCR or whole-genome amplification, in-droplet barcoding, and pooled sequencing. The 16S rRNA gene amplicon approach provides a rapid and cost-effective means to profile the taxonomic composition of all droplets in the library, enabling the identification of droplets containing potentially novel taxa without sequencing the entire genome of every droplet. For droplets identified as harboring potentially novel or functionally interesting lineages, whole-genome sequencing can be applied to recover high-quality single-amplified genomes (SAGs), allowing robust taxonomic assignment through average nucleotide identity (ANI) and digital DNA-DNA hybridization (dDDH) analyses, as well as functional characterization. Technologies such as Microbe-seq (56) have demonstrated the feasibility of recovering thousands of SAGs from complex microbial communities, achieving strain-level resolution. Another approach could be label-free single-cell Raman spectroscopy, which offers a complementary route for functional screening directly from droplet-encapsulated cells. Without the need for lysis, probes, or amplification, Raman spectroscopy detects intrinsic biochemical fingerprints that reflect metabolic activity, enabling the identification of droplets containing cells with desired functional traits, as demonstrated previously (57). These efforts will be essential to further improve the applicability of MDT to complex natural microbiomes.

In addition to these experimental design constraints, we also observed technical variability in CFU recovery rates on traditional plates between batches (e.g., ~0.04% in the preliminary experiment, versus much lower rates of 0.002–0.01% in the subsequent parallel CPM experiment). This discrepancy can be attributed to technical variability: microscopic cell counting with a hemocytometer provides only an estimate, and colony counting on plates may be compromised by overlapping colonies and fungal contamination. Therefore, we emphasize that the absolute CFU number and its recovery rate relative to the total cell number should not be interpreted as a strict measure of culturability nor compared directly across batches. Instead, these values serve as a rough reference. Nevertheless, although the recovery percentages differ, this variation does not affect our major conclusions regarding the relative performance of the two approaches.

A common challenge of MDT is that physical isolation into droplets inevitably disrupts *in situ* microbial interaction networks, including syntrophy, cross-feeding, and quorum sensing. Thus, the isolation effect of MDT is a double-edged sword: on the one hand, it releases slow-growing taxa suppressed by competition; on the other hand, it may prevent the growth of taxa strictly dependent on metabolic interactions. Nevertheless, under the oligotrophic conditions commonly observed in polar environments, competition rather than metabolic cooperation could be the primary force structuring microbial communities (58–60). Theory and empirical evidence from diverse oligotrophic systems indicate that when nutrient availability is chronically low, k-selected oligotrophs consistently outcompete r-selected copiotrophs for scarce resources by maintaining high-affinity uptake systems and low metabolic maintenance costs (58, 59). Consequently, the potential competitive release afforded by droplet confinement may outweigh the loss of potential community interactions, which could help explain the markedly improved recovery of diversity, quantity, and novelty observed in this study. However, the higher cell input group (DropAS_H) showed higher taxonomic novelty than the lower input group (DropAS_L), suggesting that multi-cell encapsulation at a moderate frequency may occasionally capture synergistic micro-colonies or metabolically interdependent cells that would be excluded under strict single-cell encapsulation. This observation hints at a promising future direction: MDT could be adapted for controlled co-cultivation by deliberately increasing multi-cell encapsulation rates (higher Poisson λ), thereby preserving beneficial metabolic interactions while still mitigating the overwhelming competitive suppression seen in bulk plate cultures.

### Conclusions

This study provides the first systematic evaluation of MDT for the isolation and cultivation of microorganisms from Arctic active-layer soils. Our results demonstrate that MDT substantially outperforms CPM across multiple metrics: recovery rates were 6.5- to 8.1-fold higher, isolation throughput improved by 180-fold, and the recovered community exhibited significantly greater taxonomic richness (256 versus 211 genera) and evenness. The near-full-length 16S rRNA gene sequencing approach enabled robust taxonomic assignment, revealing that approximately 50% of MDT-recovered sequences represented potential novel species and 27% represented potential novel genera. The differential enrichment of *Caulobacter* and other oligotrophic-adapted genera in MDT but not in CPM highlights that droplet confinement can alleviate the competitive dominance of fast-growing copiotrophs, thereby releasing slow-growing and rare taxa that are refractory to standard cultivation.

In future research, expanding the repertoire of cultivation media (e.g., with soil extracts (28) or chemolithoautotrophic media) and incubation conditions will be essential to fully exploit the advantages of MDT and further enhance recovery efficiency and taxonomic coverage. Concurrently, addressing the downstream bottlenecks of strain dereplication and targeted sorting from thousands of droplets remains a critical challenge. We anticipate that integrating MDT with culturomics and multi-omics will accelerate the construction of comprehensive polar strain collections, advancing both fundamental understanding of microbial life under extreme conditions and the biotechnological exploitation of cryospheric resources.

## Data Availability

The PacBio raw sequencing reads have been deposited in the NCBI Sequence Read Archive (SRA) under BioProject accession number PRJNA1506897.
